# Influence of Concentration on Release and Permeation Process of Model Peptide Substance-Corticotropin-From Semisolid Formulations

**DOI:** 10.3390/molecules25122767

**Published:** 2020-06-15

**Authors:** Wioletta Siemiradzka, Barbara Dolińska, Florian Ryszka

**Affiliations:** 1 Department of Pharmaceutical Technology, Medical University of Silesia in Katowice, School of Pharmacy with the Division of Laboratory Medicine, 41-200 Sosnowiec, Poland; bdolinska@sum.edu.pl; 2 “Biochefa” Pharmaceutical Research and Production Plant, 41-200 Sosnowiec, Poland; f.ryszka@biochefa.pl

**Keywords:** ACTH, ointments, hydrogel base, release, permeation, rheology

## Abstract

The transdermal route of administration of drug substances allows clinicians to obtain a therapeutic effect bypassing the gastrointestinal tract, where the active substance could be inactivated. The hormonal substance used in the study-corticotropin (ACTH)-shows systemic effects. Therefore, the study of the effect of the type of ointment base and drug concentration on the release rate and also permeation rate in in vivo simulated conditions may be a valuable source of information for clinical trials to effectively optimize corticotropin treatment. This goal was achieved by preparation ointment formulation selecting the appropriate ointment base and determining the effect of ACTH concentration on the release and permeation studies of the ACTH. Semi-solid preparations containing ACTH were prepared using Unguator CITO *e/s*. The release study of ACTH was tested using a modified USP apparatus 2 with Enhancer cells. The permeation study was conducted with vertical Franz cells. Rheograms of hydrogels were made with the use of a universal rotational rheometer. The dependence of the amount of released and permeated hormone on the ointment concentration was found. Based on the test of ACTH release from semi-solid formulations and evaluation of rheological parameters, it was found that glycerol ointment is the most favourable base for ACTH. The ACTH release and permeation process depends on both viscosity and ACTH concentration. The higher the hormone concentration, the higher the amount of released ACTH but it reduces the amount of ACTH penetrating through porcine skin.

## 1. Introduction

Corticotropin (ACTH) is a hormone secreted by the anterior pituitary gland. It is a polypeptide consisting of 39 amino acid residues with a 4.5 kDa molecular weight [1,2,3]. It has 63 hydrogen donors and 68 hydrogen acceptors. ACTH is more stable in acid solution, pH is between 4.65 and 4.8, and its isoelectric point is 8.5 [1]. Corticotropin has been used in medicine since 1952 in the form of injections. Currently, this hormone is an alternative treatment for multiple sclerosis in patients with or without corticosteroid intolerance. Proopiomelanocortins (prohormones) and corticotropin regulate anti-inflammatory and immunomodulating processes in the course of sclerosis multiplex. Recent research has shown that ACTH therapy delays the progression of the disease and exacerbates its symptoms [4,5]. Stress activates microglia and mast cells by releasing neuropeptide neurotensin and corticotropin-releasing hormone. Exacerbation of multiple sclerosis as a result of stress may be caused by dysfunction of the hypothalamic-pituitary-adrenal axis (HPA) due to decreased production of adrenal steroids [6]. Corticotropin is also used to treat rheumatoid arthritis, gout, psoriasis and ulcerative colitis [7]. Corticotropin can also be used in Crohn’s disease, bronchitis, uveitis, epilepsy, subocidopathy, idiopathic membrane nephropathy, sarcoidosis and diabetes [7,8,9,10,11,12,13]. Corticotropin, by affecting osteoblasts, increases collagen synthesis and supports the differentiation, activity and function of osteoblasts [14]. The concentrations of corticotropin-releasing hormone (CRH) and corticotropin in cerebrospinal fluid are reduced in patients with Alzheimer’s disease [15].

In market, three preparations are available containing corticotropin in doses of 0.5 mg and 1.0 mg (0.01–0.015 mg/kg/day) [7,16,17,18]. H.P. Acthar^®^ Gel, contains purified pig corticotropin and is administered by subcutaneous or intramuscular injection. This preparation is used in the treatment of, among others: West’s syndrome, bending attacks and mental retardation, usually diagnosed in the first year of life, an exacerbated form of multiple sclerosis, allergic conjunctivitis, ulcerative colitis, lupus erythematosus and vesicular epithelial separation [7,8]. The second preparation, Cortrosyn^TM^, is a synthetic form of ACTH, which contains the sequences of 24 amino acid residues of this hormone, which is only used for diagnostic purposes of adrenal insufficiency. Another preparation, Synacthen^®^ Depot, like Cortrosyn^TM^, contains initial 24 residual amino acid ACTH sequences, but it has a form of prolonged action and is used for both diagnostic and therapeutic purposes in the short-term treatment of patients in whom steroid hormones are not well tolerated or have not proven effective [7,19].

ACTH preparations are administered in the form of subcutaneous and intramuscular injections [8,20]. The cost of producing these preparations is very high, which significantly limits their use, in addition to being not well-tolerated by all patients. It is suggested that the alternative route of administration of protein-peptide substances may be semi-solid forms of the drug, applied directly to the skin of the patient [21]. ACTH is a substance which, when applied to the surface of the skin, should penetrate the deeper layers of the skin and the circulatory system, causing systemic effects. The transdermal route of administration of therapeutic substances allows obtaining a therapeutic effect bypassing the gastrointestinal tract, where it may be inactivated (e.g., protein-peptide substances) and is also much cheaper and painless There are reports of penetration and absorption of peptide substances by natural membranes. It has been demonstrated that the absorption and penetration of prolactin through the small intestine of sucking piglets is possible and it depends prolactin concentrations and the other agents (trehaloza) [22]. It has also been reported that large molecular weight peptide substances such as insulin, corticotropin, prolactin and albumin penetrate the pericardium [23]. The penetration of a substance through the membranes depends not only on its molecular weight but also significantly on its physicochemical properties.

There are no reports of release and permeation of ACTH from ointment formulations. So far only one semi-solid form containing ACTH has been tested [24]. In this study, the emulsion ointments have been prepared on the basis of creamy Lekobaza^®^ base with amphiphilic properties, containing corticotropin in various concentrations. Physicochemical parameters of ointment preparations such as pH, spreadability, rheological properties were evaluated and in vitro release test was carried out. The analysis of rheological properties and texture of the ointment showed the dependence of the tested parameters on the ACTH content in the ointment. The results of the release test showed that ACTH is released the fastest from the preparation with the lowest concentration. Higher concentration may ensure prolonged release process [24]. A very short time of hormone release from the all formulations was also found. Lekobaza is a very complex medium, contains many components, which does not give too much possibilities to modify its composition in order to increase availability of ACTH from the ointment as well as to increase time of activity.

The objective of this study was to examine various ointment media for their suitability for obtaining effective skin preparations containing ACTH. This aim was achieved by preparation of the ointment using three different media: Lekobaza^®^ Lux (oleogel), Eucerin Ointment I (anhydrous absorption medium) and Glycerol Ointment (hydrogel). Then the effect of ACTH concentration (15 mg/g or 20 mg/g or 25 mg/g) on the amount of released ACTH in the release study from prepared semi-solid formulations was determined. Based on the release test the availability parameters of ACTH from ointments were determined.

## 2. Results and Discussion

### 2.1. Selection of the Base and Preparation of the Ointments with ACTH

Three bases were selected for the study-Eucerin Ointment type I (Fagron, Kraków, Poland, Lekobaza Lux (Fagron, Kraków, Poland) and Glycerol Ointment-FP VI [25]. Eucerin I Ointment (FP XI)-anhydrous, absorptive medium, consists of cetyl stearic alcohol, lanolin alcohols and white petrolatum [26]. The aqueous number of this medium is about 300, which allows the introduction of significant amounts of water and obtaining a stable emulsion. Lekobaza Lux-multi-component, hydrated lipophilic base-gives a cooling effect, pH close to the skin condition. Thanks to the presence of water (65%) and glycerol it has an intensive moisturizing effect. It is used as a water-repellent cream base and is difficult to wash off with water. A glycerol ointment is an example of a hydrogel-like base described in the Polish Pharmacopoeia VI as Glycerol Unguentum. The swelling agent in a mixture of glycerol, water and gelling is in this medium wheat starch. The soft and porous structure and high-water content means that hydrogels can be carriers of many drugs, including peptides and proteins with the possibility of ensuring a constant release over a limited period of time-controlled swelling behaviour. In addition, this porosity can be controlled by changing the density of the gel matrix cross-linking. The release rate (another important parameter for drug carriers) depends mainly on the diffusion coefficient of this molecule through the gel network and can also be adjusted according to the specific requirements. The hydrogel aqueous environment can stabilise cells and complex drugs, including peptides, proteins, oligonucleotides and DNA. The modification of the composition of the hydrogel base used may allow obtaining a base for the controlled release of active substances from the formulations prepared [27,28,29,30,31,32].

Investigators suggest that ointments prepared using Unguator, regardless of the composition, give a higher diffusion coefficient than ointments prepared using the conventional technique (in a mortar) [33]. In this study the ACTH ointment mixing parameters were selected in such a way that the mixing time and agitator rotation speed did not significantly affect the rheological parameters. Too high a turnover can change the viscosity of the preparation. Although according to Patere et al., the rheological properties of finished products are influenced to a greater extent by the quality and origin of the raw materials used than the ointment method itself. They used 2 methods: (1) 2100 rpm 9 min, 970 rpm 1 min and 810 rpm 1 min plus with resting time 5 min and (2) 1130 rpm 9 min, 970 rpm 1 min, 810 rpm 1 min and resting time 5 min [34].

### 2.2. Evaluation of the Appearance and Uniformity of the Ointments

**Figure 1:**
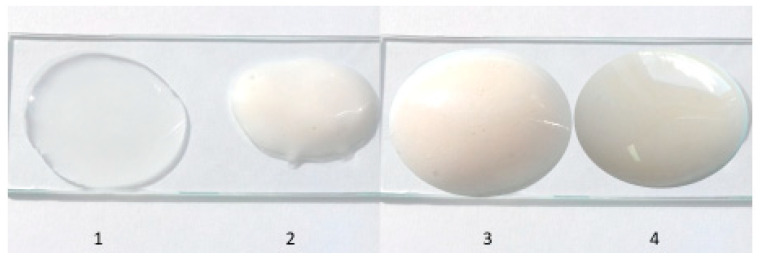
Macroscopic image of the hydrogel ointment and prepared ointments with ACTH: 1-Glycerol ointment, 2-Glycerol ointment with ACTH 15 mg/g (F-9), 3-Glycerol ointment with ACTH 20 mg/g (F-8) and 4-Glycerol ointment with ACTH 25 mg/g (F-7).

The base-glycerol ointment and sample ointments containing ACTH are presented in Figure 1. The glycerol ointment is a transparent hydrogel, and the addition of the active substance has changed the appearance and colour of the ointments made. Prepared ointments with ACTH (F-7-F-9) were not transparent, but they were from milky cream to milky beige. The colour of the ointment was related to the ACTH amount, the more the hormone, the darker the ointment colour. Ointment with 15 mg/g ACTH concentration was creamy, 20 mg/g ointment was dark creamy and 25 mg/g ointment was milky beige. No solid particles or droplets of water were found in the ointments; corticotropin was completely dissolved in the ointment base used. The homogeneity studies of the obtained ointment formulations with ACTH indicate that the use of a prescription mixer in the preparation of the ointment allowed for homogeneous dispersion of the drug substance in the ointment base and obtaining homogeneous preparations.

### 2.3. Ointment pH Measurement by Potentiometric Method

Table 1 shows the results of pH measurements. Measurements were taken for Glycerol Ointment (F-control) and for obtained ointment containing ACTH: 15 mg/g (F-9), 20 mg/g (F-8) and 25 mg/g (F-7).

The pH value for Glycerol Ointment was 4.96. Because of alkaline character of corticotropin, its addition to ointment base increased this value respectively to its concentration increase. The higher concentration of ACTH is the more pH value. The lower pH value was observed for F-9 formulation (15 mg/g ACTH)-5.99 and the highest pH value-for F-7 formulation (25 mg/g ACTH)-6.44. The values obtained ointment formulations are suitable for physiologic skin pH values-4.5–6.5. So the ointments can be safely applied to the skin.

### 2.4. Corticotropin Release Study from the Hydrogel Ointments

For testing the release of substances in vitro, three different methods are recommended using: (1) USP 4 with semi-solid adapters; (2) USP 2 apparatus with Enhancer cells; and (3) Franz diffusion cells. Bao et al. examined the release from eye ointments, they found that for some substances, USP 2 and USP 4 are more suitable than the Franz chambers method for the better reproducibility of the results. The high-quality design of cells or adapters for sampling (Enhancer cells and semi-solid adapters) ensure good reproducibility of the sample placement. In addition, pharmacopoeial methods were standardised [35,36].

To determine the release of ACTH, USP 2 apparatus with the Enhancer cells was used. Artificial cellulose membranes were used in the release study in order to obtain the repeatability of the determinations of the amount of released ACTH from the ointment.

It is suggested that the release process depends on the properties of the membrane through which the drug substance diffuses into the acceptor fluid. It has been reported that artificial cellulose membranes give a higher reproducibility of results than natural porcine membranes due to the method of preparation of the natural membrane. Porcine membranes, due to their lipophilic nature, may be better permeable to some substances, whereas artificial films may be more beneficial to other substances [37,38,39,40,41]. The porcine membrane-the skin of the ear-was used for ACTH permeation study.

Olejnik et al. in release studies of undecylenoyl phenylalanine from topical formulations used various synthetic membranes, such as regenerated cellulose (Cuprophan), nitrocellulose, cellulose acetate, cellulose acetate and membrane imitating human skin (Strat-M). Since the kinetics of release may also be affected by the composition of the preparations, the release of undecylenoyl phenylalanine from semi-solid formulations was performed by one selected membrane from regenerated cellulose - Cuprophan, for which the highest correlation coefficients were obtained [42]. So, in this study the artificial membranes from regenerated cellulose were used in the ACTH penetration test to obtain repeatability of the determination of the amount of released ACTH from the ointment.

**Figure 2:**
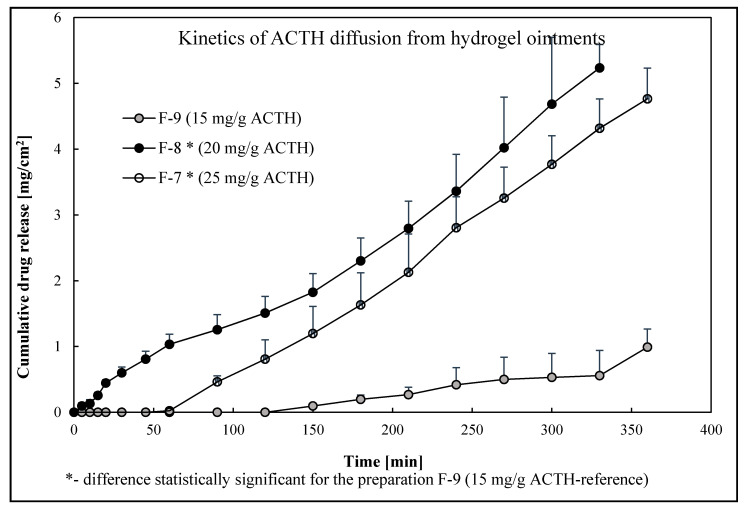
In vitro dissolution profiles at 32 °C-cumulative drug release.

In the release study of ACTH from ointments made on the basis of Lekobaza Lux (F-1-F-3) and Eucerin ointment I (F-4-F-6), no ACTH release was observed within six hours. Assuming that such a small amount of ACTH was released from Eucerine ointment I and Lekobaza Lux that the analytical technique proved to be insufficiently sensitive, it would make no use of preparing such ointments. It would be difficult to achieve the therapeutic concentration and thus the effectiveness of the semi-solid form of the drug. Therefore, further research was omitted; the Eucerin ointment I and Lekobaza Lux ointment were therefore rejected as a potential support for ACTH. The differences and no release of ACTH from Eucerin I Ointment and Lekobaza Lux could be explained by the different physicochemical parameters of the ointment bases and its solubility in water. However, the release of corticotropin from the Glycerol Ointment (hydrogel) medium (F-7, F-8, F-9) was observed. The cumulative amount of released ACTH from the hydrogel base during 360 min is shown in Figure 2.

As shown in Figure 2, the largest amount of ACTH was released from the F-8 formulation with a concentration of 20 mg/g-5.23 ± 0.36 mg/cm^2^, from the F-7 formulation with a concentration of 25 mg/g was 4.77 ± 0.47 mg/cm^2^ and the smallest of the F-9 with a concentration of 15 mg/g-0.99 ± 0.27 mg/cm^2^. The delayed release of ACTH associated with a higher time-lag may be related to the affinity of the hormone to the ointment base.

The ACTH formulation of 15 mg/g is most strongly associated with the ointment base. The higher the concentration of ACTH in the ointment, the more the active substance penetrates into the acceptor (R^2^ = 0.811). The obtained results confirmed the desirability of using an ointment of hydrogel ointment-glycerol ointment to obtain semisolid formulations with ACTH.

The Weibull method (*p* < 0.05) showed significant statistical differences in the ACTH release profiles from the prepared ointments (fitting coefficients: F-7, R^2^ = 0.521 and F-8, R^2^ = 0.844) in relation to the F-9 formulation.

#### 2.4.1. Kinetics Calculations

Based on the investigation of Xu et al., it was found that kinetics and the mechanism of drug release are highly dependent on the type of ointment bases. In oleaginous bases, drug release following a unique logarithmic-time dependent profile; in both absorption and water-soluble bases [43].

For analysis of the permeation data, the amounts of drug permeated are plotted against the time. In many cases, an excess of drug is applied to the skin surface (infinite dose), and its depletion is negligible. In these cases, drug permeation follows zero-order kinetics, which characterizes the drug diffusion across the skin according to Fick’s law. In these cases, a linear equation is calculated in the steady state, and its inclination represents the flux of drug across the skin, also denominated as the permeation rate. In contrast, when a finite dose of drug is applied to the skin, the relationship between amounts of drug permeated and time is not linear. When the drug amounts applied to the skin decrease along the time, the permeation rate is also decreased. Drug flux through the skin under non-saturated conditions follows pseudo-zero order kinetics (or Higuchi kinetics), in which the drug concentration is proportional to the square root of time [44,45,46].

The release results were fitted to various kinetic models: zero order kinetics, firs order kinetics, Higuchi model and Korsmeyer-Peppas model. In the zero-order model-to investigate the kinetics of release-data obtained from in vitro studies are presented as the cumulative amount of drug released in relation to time. This is used in the evaluation of release from modified dosage forms as in some transdermal systems, as well as from matrix tablets with weakly soluble drugs in coated forms, from osmotic systems with controlled release process [47,48,49].

The first order model may be used to describe the drug dissolution in pharmaceutical dosage forms such as those containing water-soluble drugs in porous matrices [47,50]. The first example of a mathematical model to describe the release of drugs from the matrix system, including semi-solid drug forms, was proposed by Higuchi in 1961 [51]. The model is based on the hypothesis that the initial drug concentration in the matrix is significantly higher than the drug solubility, the drug diffusion take place only in dimension, drug molecules are much smaller than the matrix thickness, matrix swelling and dissolution are negligible, the drug diffusivity is constant and perfect sink conditions are always attained in the release environment. The data obtained were presented as cumulative percentage drug release versus square root of time [50]. The model can be used to characterise the drug dissolution from several types of modified release pharmaceutical dosage forms in the case of some transdermal systems and matrix tablets with water soluble drugs [47,51,52,53]. The Korsmeyer-Peppas model derives from a simple relationship which described drug release from a polymer matrix [47,54]. To study the release kinetics, data obtained from in vitro drug release studies were plotted as log cumulative percentage drug release versus log time.

Weibull’s model has been described for different dissolution process. This model is more useful for comparing the release profiles of matrix type drug delivery, to establish statistical differences in the release profiles [47,55].

In the ACTH ointment release study, R^2^ values were calculated for each model. For all studied formulations the highest correlation coefficient was observed for the Korsmeyer-Peppas model (Table 2), the others were as follows: K-P model (logaritmic) > zero-order > Higuchi > first-order kinetics. The chosen model seems to be suitable for the release of ACTH from the ointment because the obtained formulation is made on the basis of hydrogel, from a natural polymer, which is starch.

The three most commonly used models (zero-order, logarithmic and Higuchi) were used to investigate the kinetics of the release of loteprednol ethabonate from ointments based on lipophilic base (white petroleum jelly and mineral oil 69.2%:30.3% (*w*/*w*)). The value of the fitting factor (R^2^) between the three models showed the following order: Higuchi (more suited for lipophilic ointments) > logarithmic > zero order [56,57].

Olejnik et al. in studies of undecylenoyl phenylalanine (Ude-Phe) release from the formulation for skin application (studies of Ude-Phe from topical formulations), depending on the membrane used, described the results with different models: For Cuprophan membrane the Higuchi model was used, for cellulose acetate and nitrocellulose membranes the Korsmeyer-Peppas model, while for Strat-M membrane the highest correlation coefficient was observed for reaction models “0” and “I” of the order. The kinetic model was also dependent on the physical and chemical form of the ointment used, whether it was a microemulsion, macroemulsion or hydrogel. Release from hydrogels followed the diffusion process, according to Fick’s law. They found out that the largest amount of active substance in the shortest time was released from the hydrogel based on carbomer with addition of isopropanol, little smaller from hydrogel based on hydroksyethylcellulose, while the smallest amount of drug was diffused from macroemulsion. This could be also explained by different viscosity of these formulations, a higher viscosity of hydrogels than hydro-alcoholic gels, better availability of Ude-Phe in hydrogel than in macroemulsion. The diffusion of active compound through oily phase might be a limiting step for drugs release [42]. The same explanation it can be applied to ACTH release from Eucerin ointment and Lekobaza Lux. Hydrogels can be better vehicle for the drugs. Lekobaza Lux as hydrophobic gel and Eucerin ointment I as an anhydrous absorbent lipophilic medium represent higher values of viscosity and it can affect the ACTH release process. Formulations based on Lekobaza Lux and Eucerin I Ointment represent emulsion system, while formulations based on hydrogel are characterized by present of soluble drug in ointment base. The diffusiveness of drug from solutions is better than from emulsions systems. It was reported that ACTH was also released from the creamy amphiphilic medium-Lekobaza. The formulations were emulsions type. From these preparation corticotropin was released the fastest from the ointment of the lowest concentration; with increasing concentration, the rate of ACTH release decreased. The highest correlation coefficient in determining kinetic models was recorded for the Higuchi model [24]. On the other hand, from all formulations based on glycerol ointment ACTH was released longer than 150 min and its amount increased with increasing concentration in the ointment. ACTH was dissolved in ointment’s base component-glycerol. The way introduction of ACTH to ointment form affected release rate and ACTH released amount. 

Various arrangements may be used to enhance percutaneous absorption of drugs that are not ideal candidates for transdermal effect based on their properties. Fick’s first law equation describes how effectively percutaneous absorption can be enhanced: by increasing the diffusion coefficient, by increasing drug partitioning into the skin and by increasing the degree of drug saturation in the vehicle. The concentration gradient and the partition coefficient will be greatly influenced by the formulation. Because of this the change permeability may be influenced by changing the design of the formulation. Increasing the drug concentration in the vehicle can provide to increasing in degree of saturation. In particular, the creation of a supersaturated solution of the drug in the vehicle is desirable, as this will generate a thermodynamic drive for the drug to diffuse out of the vehicle and into the skin [58].

Selection of the studies method must be performed by using experimental conditions, such as skin model, experimental apparatus, and receptor medium, appropriate for the formulation to be evaluated, and it must be based on the physicochemical properties of the drug, including aqueous solubility and oil-water partition coefficients. ACTH is a water-soluble substance with an isoelectric point within 8.5 and a lipophilicity log P = 4.9.

The lipophilicity of some substances, such as (ACTH antagonist), can certainly constitute a significant obstacle to acceptable solubility and pharmacokinetic properties, such as brain penetration. The calculated log P of antalarmin (corticotropin-releasing factor antagonist) is very high (7.0) and straight chain alkyl groups significantly impact the overall lipophilicity of these molecules. The most common strategy utilised to lower the lipophilicity of the top region substituent is oxygenation. Hydroxylation of the butyl group in compound lowered the overall lipophilicity (log P = 5.2) without compromising CRF1 affinity and the exchange of methylene moieties for oxygen led to the diether analogue, (log P = 4.3) [59].

Several recent studies have focused on the development of transdermal forms for administration of donepezil, an anti-Alzheimer drug [60,61,62,63]. In these studies, the permeation of this lipophilic compound (log P > 4) was studied by using different experimental protocols, including skin models, experimental apparatus and formulations. Choi and co-workers [60] demonstrated a 1-fold reduction in the results of donepezil permeation by using human cadaver skin relative to the results of studies using hairless mouse skin, a skin model with increased permeability. Liu et al. [61] used rabbit abdominal skin, Subedi and co-workers [62] used isolated hairless mouse skin. These authors also achieved satisfactory results by using these skin models; however, the amounts of drug permeated were overestimated in relationship to permeation across normal human skin [44,60,61,62,63]. Comparison of skin permeation data from different studies is not a simple task, and permeation data must be analysed to determine the effects of the formulations under the same experimental conditions. In this way, it is possible to identify the variables in formulations that increase drug flux through the skin as a way to select promising formulations for evaluation in clinical trials [44].

#### 2.4.2. Pharmaceutical Availability of ACTH from Obtained Ointments

Table 3 presents the pharmaceutical availability of ACTH from semi-solid formulation: the size of the area under the concentration-time curve (AUC_0-6h_) and the degree of relative availability (EBA, %).

The highest pharmaceutical availability is characterized by the formulation (F-8) containing 20 mg/g of ACTH. Availability of ACTH from ointment F-8 is approximately 10 times higher than from ointment 15 mg/g (F-9). A positive correlation between the hormone concentration in ointment and the pharmaceutical availability was found (R^2^ = 0.683).

Analysing the results of the pharmaceutical availability test, it can be concluded that corticotropin is released to a greater extent from ointments with higher hormone concentrations (R^2^ = 0.812). The higher pharmaceutical availability results in a shorter time of action of the preparation, while ointments of lower concentration may show a prolonged release process.

### 2.5. Skin Permeation Study

**Figure 3:**
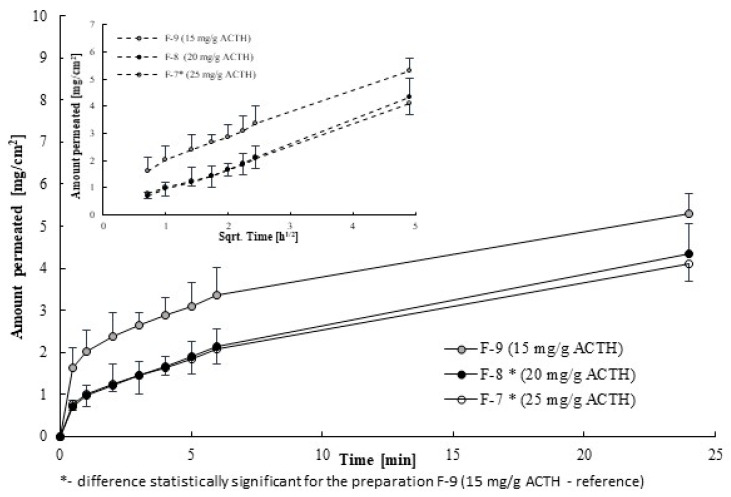
Kinetics of ACTH permeation through the porcine skin.

Based on the permeation test it was found that ACTH penetrates through the porcine skin from all tested ointments. As shown in Figure 3, the amount of coricotropin permeated across the skin was increasing with time from hydrogel ointments. According to Figure 3, even though the cumulative amount was increasing with time, the rate of ACTH permeation was actually reducing with time.

Within 24 h it penetrates 6.95 ± 0.54% (F-7-25 mg ACTH per 1 g of ointment), 9.30 ± 1.31% (F-8-20 mg ACTH per 1 g of ointment and 15.07 ± 2.71% (F-9-15 mg ACTH per 1 g of ointment/g, respectively. The results obtained suggest that the degree of corticotropin penetration through the skin may be related to their concentration in the ointment. The higher concentration of corticotropin in the ointment, the lower degree of its penetration through the skin.

A high negative correlation was observed. (R^2^ = −0.9717). Increasing the loading of ACTH in the hydrogel bases, with using natural membrane-porcine ear skin, did not promote drug permeation into the acceptor fluid. The most amount of ACTH permeated from ointment with the smallest concentration. In case ACTH release from ointment into the acceptor fluid using artificial membrane the opposite dependence were obtained.

As shown in Table 4, the rate of penetration decreases with the loading of ACTH. The fastest ACTH permeates from the ointment with the lowest concentration (*p* < 0.05)-15 mg/g (F-9), about 1.3 times faster than from the ointment with the highest concentration-25 mg/g (F-7). From F-7 formulation ACTH penetrates the slowest. 

### 2.6. Rheological Properties of ACTH Ointments

Viscosity is not a constant value and depends on factors such as temperature and shear rate. The temperature for storage or application of the preparation can be used for measurements. The range of shear rate can also be selected based on the storage of the ointment or the technique of spreading it on the skin [63]. The viscosity of ointment formulations at 25.0 °C at two shear rates was determined: 15 s^−1^ and 30 s^−1^. The determined values of viscosity and shear stress are presented in Table 5.

The addition of ACTH causes an increase in viscosity in comparison to not loaded base (F-control). Both of at the shear rate D = 15 s^−1^ and D = 30 s^−1^ this increase is noticeable in all formulations containing ACTH. 

The lowest viscosity (η) was observed for ointment not loaded with active substance (F-control, η = 6.01 ± 0.26 Pa·s at D = 15 s^−1^ and η = 6.08 ± 0.17 Pa·s at D = 30 s^−1^). The highest viscosity for ointment containing ACTH at concentration of 25 mg/g (F-7) was observed. The results of the viscosity determination clearly show that the viscosity increases with increasing ACTH concentration in the ointment.

Wróblewska et al. developed a technology of preparation and optimal composition of hydrogels with sulphur using various types of polymers such as hydroxyethylcellulose (HEC), Carbopol 980 and sodium alginate. Obtained hydrogel formulations containing sulfur were found to have different viscosity values than these unloaded. The highest viscosity was noticed in the case HEC hydrogel (12,144 mPa·s at a shear rate of 10 s^−1^) and the lowest in the case of Carbopol 980 hydrogel (6582 mPa·s at a shear rate of 10 s^−1^). The addition of sulphur increased the viscosity, which was particularly noticeable in the formulations obtained from HEC [64]. Regardless of whether the active substance occurs in solid form as described by Wróblewska or dissolved formulations as in the case of corticotropin, it often increases viscosity. 

The flow test was carried out in the shear stress range 5.0–200.0 s^−1^. The results were presented as dependence of shear stress on shear rate in a form of flow curves as well as hysteresis loops in a step-by-step test. The measurement time during flow test was 30 s. The ascending curve time from 5–200 s^−1^ was 6.5 s, the descending curve time from 200 to 5 s^−1^ was 9.7 s. The measurement time for the step-by-step test was 240 s.

**Figure 4:**
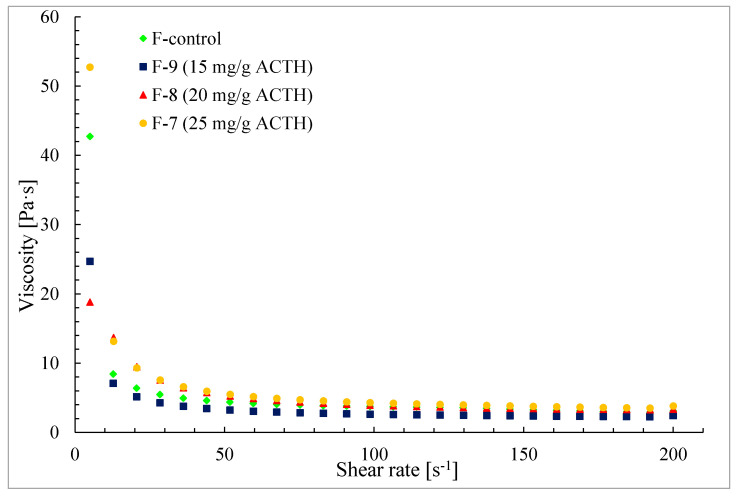
Viscosity curves of hydrogel F-7–F-9 and F-control (Glicerol ointment).

**Figure 5:**
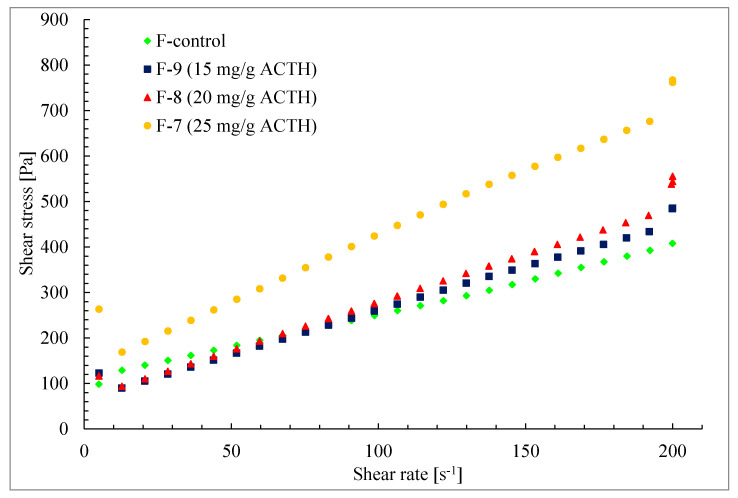
The flow-curves plotted for the ointments F-7–F-9 and F-control in the controlled stress mode.

In all ointments obtained, containing ACTH it was shown that with increasing shear rate the ointment viscosity decreases (Figure 4). The systems are diluted with shear rate, belong to thixotropic systems and confirm these properties by the shape of viscosity curves in Figure 5.

Thixotropy is the property exhibited by the pseudo plastic systems which exhibit the time-dependent change in the viscosity. Thixotropic systems demonstrate a decrease in viscosity with time under the constant shear. An enhancement of shear due to progressive breakdown of the structure of liquid and further rebuilding of the structure due to Brownian motion, which makes the particles move to their most favorable positions from a structure-entropy perspective, is assumed to be the reason for pseudo plasticity [65].

The relationship between shear stress and shear rate is presented in Figure 5. The course of the flow curves of all prepared ointments indicated a non-Newtonian character. The relationship between shear stress and shear rate deviated from a straight line, and there was also an offset along the ordinate in the shear rate range from 5 s^−1^ to 200 s^−1^.

Therefore, the equation for non-linear viscoelastic bodies, Casson’s Equation (1), was used to approximate the values of shear stress and shear rate:

(1) $$\sqrt{\tau }=\sqrt{\tau_{y}}+\sqrt{\eta \gamma }$$

where τ = shear stress [Pa], τ*_y_* = constant interpreted as yield stress [Pa], η = experimentally determined viscosity [Pa·s] and γ = shear rate [s^−1^]. This method allows the determination of the flow limit, and its existence is indicated by the shape of the flow curve.

There are a number of ways to quantify the thixotropic behavior of materials. The most often the measure the area within the hysteresis loop from a shear rate curve and viscometers or rheometers measures to assess rheological behaviour at varying shear stress and shear rates are used. The most suitable way for the measurement of thixotropy is to describe the material response in shear stress due to an inflicted deformation or a shear rate during flow test [64]. The shear rate increased with time until it reaches a maximum shear value. Thereafter, without any disturbance, the process is reversed by decreasing the shear rate, leading to the formation of up and down curves. The area enclosed by the up and down curve is referred to as hysteresis loop. The hysteresis loop approach is at present the most widely used method and was successfully utilized in the studies of the thixotropic behaviour of various hydrogels and various its concentrations.

**Figure 6:**
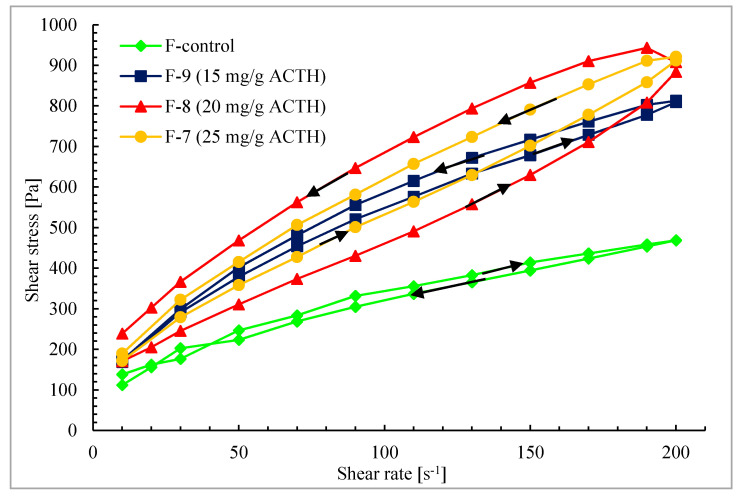
Hysteresis loops for formulation F-7–F-9 and control ointment-F-control.

The ability to return to the initial structure of the hydrogel indicates its rheological stability. The unloaded hydrogel (F-control) shows the lowest degree of deformation due to shear stress. The hysteresis loop created by this ointment covers the smallest area. The ointment with the lowest ACTH concentration of 15 mg/g (F-9) proved to have the most similar shape and surface area of the hysteresis loop to a unloaded ointment (F-control) (Figure 6). Ointment with the highest amount of ACTH-25 mg/g (F-7) shows lower stability for deformation in Flow-test in comparison to F-9 (with the lowest amount of ACTH).

The ointment with 20 mg/g (F-8) shows the largest area of the hysteresis loop, so it can be concluded that in this case the return to the initial structure will require the most time.

Referring to the results of ACTH release from ointments, it should be stated that the largest amount of ACTH was released from F-8 ointment containing 20 mg/g of ACTH, characterized by the largest area of hysteresis loop. It can be assumed that ACTH in this formulation is the least related to the base, which is proved by a larger area. This may be related to the solubility of corticotropin and for this reason a higher concentration of 25 mg/g does not give such good results as a concentration of 20 mg/g ACTH. The release process of ACTH depends mainly on the concentration in the ointment, but also on viscosity. As the hormone concentration increases, the amount of released ACTH increases.

In the case of higher concentrations it seems that the higher viscosity decreases the released amount of ACTH. With an increase in concentration from 20 to 25 mg/g the viscosity increases, but the amount of ACTH released is lower.

Ointment with concentration of 15 mg/g ACTH (F-9) is characterized by the highest recovery and the diffusion coefficient for this formulation is much lower than for F-8 formulation with higher concentration of ACTH.

Viscosity may directly influence the diffusion rate of drug at the microstructural level. Yet semisolid drug products with high viscosity still can present high diffusion rates compared to semisolid products of comparatively lower viscosity. These observations emphasize the importance of rheological properties of semisolid dosage forms, specifically viscosity, on drug product performance. Rheological properties of ointments have a great significance during production process (e.g., filling of tubes, pumping through tubes, and storage), application from the container to the administration place (the ointment should not be too thick or too thin) and aesthetics. In addition, rheological properties are critical to understand the microstructure of ointment formulations [56,66,67].

## 3. Materials and Methods

### 3.1. Materials

ACTH, a peptide hormone with the chemical formula C_207_H_308_N_56_O_58_S, is isolated from porcine hatches in a lyophilised form which is easily soluble in water (Biochefa, Sosnowiec, Poland). 

Two ointment media were used in the study, which are pharmaceutical raw materials intended for the preparation of semi-solid preparations for use on skin or mucous membranes. The third medium was prepared on the basis of a recipe in Polish Pharmacopoeia VI [25]. 

Ointment bases-Lekobaza Lux (Fagron, Kraków, Poland)-pharmaceutical material, Eucerin ointment I (Fagron, Kraków, Poland)-pharmaceutical material and glycerol ointment-Glyceroli Unguentum composed of wheat starch (10.0), purified water (15.0), glycerol 85% (90.0) and ethanol 760 g/L (1.0).

Excipients: 1 M acetic acid (POCH, Gliwice, Poland), wheat starch (Galfarm, Kraków, Poland), glycerol 85% (Galfarm, Kraków, Poland), ethanol (Alpinus Chemistry, Solec Kujawski, Poland), purified water (Fagron, Kraków, Poland), NaCl (Chempur, Piekary Śląskie, Poland), disodium hydrogen phosphate (Avantor Performance Material S.A., Gliwice, Poland) sodium hydroxide (Avantor Performance Material S.A.). The substances used were of the PD class and meet the requirements of standards for pharmaceutical raw materials.

### 3.2. Preparation of Ointment with ACTH

The ACTH ointment formulations with concentrations of 15 mg/g, 20 mg/g or 25 mg/g were prepared using three ointment bases. One of the One of the bases was a hydrated lipophilic absorption medium, Lekobaza Lux (Ointments F-1, F-2 and F-3). The second base was Eucerin ointment I as an anhydrous absorbent lipophilic medium (Ointments F-4, F-5, F-6). The third base was glycerol ointment-Glyceroli Unguentum [25] (Ointments F-7, F-8, F-9).

Ointments containing ACTH were prepared using Unguator CITO e/s (Eprus, Bielsko-Biała, Poland). When preparing ointments based on the Eucerin I Ointment, the active substances were emulsified into the medium as an aqueous solution. For this purpose, defined (150.0 mg, 200.0 mg and 250.0 mg) amounts of corticotropin were weighed and dissolved in 1 mL of aqueous 1 M acetic acid. To a tared container with a capacity of 33 mL, 9 g of ointment base was weighed and then stirred with Unguator at level 5 (1630 rpm) for two minutes. Mixing parameters-time and level of rotation-were determined on the basis of a preliminary experiment. Parameters depend to a large extent on the amount of the preparation. For larger samples the mixing time should be longer. Incorrectly selected rotations also affect the quality of the product. Too high a speed can cause excessive air bubbles. The mixed ointment base (about 8.0 g) was transferred to another tared container, then corticotropin solution (150.0, 200.0 and 250.0 mg in 1 mL of acetic acid was added and supplemented to 10.0 g with the base. The ointment was mixed again on 5 levels (1630 rpm) for 2 min.

In the case of making an ointment based on a glycerol ointment, the ointment base was first made. The glycerol ointment was prepared according to PPh VI ed., 2002 [25]-wheat starch was mixed with water, then glycerol was added in the portions and mixed. The whole was heated in a water bath (AJL Electronic MLL 147, Kraków, Poland) to create a uniform, transparent mass. The ointment was transferred to a container, ethanol was added and homogenised with Unguator for two minutes at level 5. The active substance was then ground with a few drops of 85% glycerol, then added to the glycerol ointment (the ointment was supplied to 10.0 g per weight) and homogenised in the Unguator at the 5th level of rotation (1630 rpm) for two minutes. Corticotropin was dissolved during mixing in Unguator. 

In case of preparation of ointment based on Lekobaza Lux, the active substance was rubbed with steichiometric amount of Glycerol ointment, then the rest of substrate was added in portions and finally homogenized using Unguator at 5 rpm for 2 min. The composition of individual ointment formulations (F-1–F-9) is presented in Table 6.

### 3.3. Macroscopic Evaluation of the Formulated Ointments with ACTH

The applied ointment bases and prepared ointments were subjected to colour observation [68]. A plate test was also carried out to assess the homogeneity of the preparations. The test consisted in applying a thin layer of ointment to the glass plate and after observing under the microscope the presence or absence of clots on the active substance, too large droplets of water or air bubbles [68]

### 3.4. pH Measurement

The specification of the drug in the form of dermatological ointment provides for pH measurement to be performed by potentiometric method (FP XI)-Polish Pharmacopoeia 11-th edition, 2017 by direct immersion of a glass electrode in a semi-solid dosage form [26]. An InLab Expert Pro-ISM electrode (Part No. 30014096, Mettler-Toledo AG, Greifensee, Switzerland) was used for the examination. The measurements were taken for the ointment formulation: Glycerol ointment (F-control), ointment containing respectively15 mg/g ACTH, 20 mg/g ACTH and 25 mg/g ACTH at the temperature 25.0 ± 0.5 °C, assumed at the storage or application temperature of the preparations on the skin. Each measurement was carried out five times and average pH was calculated. pH values are presented as the mean ± standard deviation (SD).

### 3.5. Methodology of ACTH Determination

A standard curve for a corticotropin solution in 0.1 mol/L acetic acid was prepared. The analytical wavelength was determined for spectrophotometric determinations. The spectrum was made in the UV range for a solution of ACTH in 1 mg/mL acetic acid. The wavelength corresponding to the maximum absorbance for ACTH was λ = 276.5 nm. 0.1 mol/L acetic acid was used as the reference. Measurements were made using UV-VIS Cecil CE 3021 spectrophotometer (Cecil Instruments Limited, Cambridge, UK).

The calibration curve was made on the basis of the absorbance of corticotropin solution in 0.1 mol/L acetic acid with the following concentrations: 0.1, 0.2, 0.4, 0.5, 0.6, 0.8 and 1.0 mg/mL. Absorbance measurements were made in quartz cuvettes with a thickness of 1 cm at the wavelength, λ = 276.5 nm. 0.1 mol/L acetic acid was used as the reference. The average mean of the five repetitions was calculated. The photometric accuracy of the spectrophotometer was ±0.005 A. The corticotropin content was calculated from a standard curve with the equation y = 0.6925x − 0.0123; R^2^ = 0.9996.

For the applied ointment bases-Eucerin ointment I, Lekobaza Lux and glycerol ointment-no interference was found at the wavelength chosen λ = 276.5 nm. It was specific only for the determination of ACTH.

### 3.6. ACTH Release from Ointment Formulations In Vitro

A modified USP Apparatus 2 equipped with 200 mL flat bottom vessels and mini paddles (Erweka DT 600, ERWEKA GmbH, Langen, Germany) was used for drug release studies. Excess amount of the ACTH ointment was loaded inside a special semisolid holder, Enhancer fluoropolymer cells (ERWEKA GmbH) with an exposed area of 3.8 cm^2^. Using a spatula, ointment surface was flattened, smoothed, and excess material removed, after which the exact loaded ointment quantity was determined by weight. As a result, 1.0 g of ointment with the exactly defined active substance content was introduced into the Enhancer cells. A pre-cut and pre-wetted a regenerated cellulose membrane Spectra/Por^®^2 Dialysis Membrane MWCO: 12-14 kDa, imitating the skin barrier (Fisher Scientific, Loughborough, UK), was placed on top the ointment before capping the enhancer cell and removing excess water from the membrane. 50 mL of phosphate buffered saline (PBS) pH 7.2 was used as an acceptor liquid. The process temperature was maintained at 32 °C throughout the study (it reproduced the temperature of the human skin). The agitation speed of the agitators was 50 rpm. The selected conditions reflected the physiological conditions prevailing in the system. The study was carried out in 360 min. Aliquots of 3 mL were collected at 17-time intervals: 5, 10, 15, 20, 30, 45, 60, 90, 120, 150, 180, 210, 240, 270, 300, 330 and 360 min. The volumes of samples that were taken for measurements were supplemented with 3 mL of PBS at 32 °C. In collected samples, the amount of released corticotropin was determined by spectrophotometric measurement. The total cumulative amount was presented as the amount released per unit area of skin [mg/cm^2^] within 360 min. The release profiles was made based on obtained results.

#### 3.6.1. Kinetics Calculations

The release results were fitted with different kinetics models such as zero order (% ACTH release vs. time), first order (log of % ACTH remaining vs. time), Higuchi’s model (% ACTH release vs. square root of time) and Korsmeyer-Peppas model (log of % ACTH release vs. log time). For each model R^2^ values were calculated.

#### 3.6.2. Determination of ACTH Availability from Hydrogels Formulations 

The availability of ACTH from the ointment (AUC_0-6h_)-the area under the concentration-time-change curve was calculated. Area under the curve of concentration changes-time was counted as a numerical integral, using the parabol method. The size of the area under the concentration-time curve (AUC_0-6h_) was used to calculate the degree of relative availability (EBA, %) of ACTH 20 mg/g and ACTH 25 mg/g of ointment relative to the ACTH 15 mg/g reference, according to Equation (2):

EBA = (AUC_ACTH 20 mg/g_;)/(AUC_ACTH 15 mg/g_) × 100% (2)

The obtained results were compared to the F-9 formulation, containing 15 mg/g of ACTH based on glycerol ointment. The existing interdependencies (R^2^) between the ACTH concentration in the ointment and the total amount of released hormone after six hours and AUC were calculated.

### 3.7. In Vitro Skin Permeation Studies

#### 3.7.1. Vertical Franz Diffusion Cells

**Figure 7:**
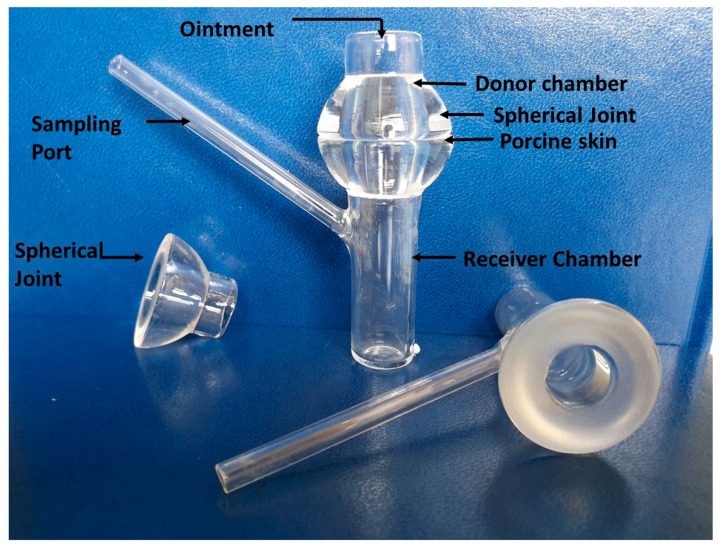
In vitro ACTH permeation study with porcine ear skin mounted on the vertical Franz cell with a spherical joint.

Franz diffusion cells (Xenometrix, Allschwil, Switzerland) with inner diameters 15 mm and permeation area of 1.77 cm^2^ were used in the permeation study (Figure 7). An accurately weighed amount of the formulation (500 mg) was placed onto the membrane in the donor compartment and the device was closed with parafilm. The acceptor compartment was filled with 12 mL of PBS pH 7.2. During the experiment the acceptor fluid was kept in water bath at 32 °C. After 0.5, 1, 2, 3, 4, 5, 6 and 24 h, 2 mL of sample was taken and the sample volumes were made up to the initial volume with 2 mL PBS at 32 °C. The amount of permeated corticotropin was determined by spectrophotometric measurement at the wavelength, λ = 276.5 nm, PBS was used as the reference. The average mean of the five repetitions was calculated. The corticotropin content was calculated from a standard curve with the equation y = 0.6925x − 0.0123; R^2^ = 0.9996. Total cumulated amount was presented as permeated amount per skin surface unit [mg/cm^2^] within 24 h.

#### 3.7.2. Skin Preparation

Porcine skin was used to study the permeation profile of ACTH due to the limited availability and difficulties associated with the use of ex vivo human skin. Nevertheless, porcine skin is a suitable alternative due to the similarities in thickness and permeability properties to human skin [69]. The model membrane for the skin permeation studies was full-thickness porcine ear skin acquired from a local stockbreeder. The skin was washed with 0.9% NaCl solution to absorbance E < 0.02 at wavelength λ = 276.5 nm and dried using laboratory tissue paper. Hair was carefully cut by scissors to avoid any damage to the stratum corneum and the subcutaneous fatty layer was removed using a scalpel. The full thickness skin samples were then wrapped in aluminium foil and stored at −20 °C. The skin sections were trimmed to disc the diameter 2.5 ± 0.5 cm^2^. Each of the formulations was applied to the skin surface.

### 3.8. Rheological Experiments

Rheological tests were performed only for ointments releasing ACTH-for all hydrogels with corticotropin (F-7–F-9) and for hydrogel unloaded with ACTH. The analysis was performed using universal Reometry RM 200 Touch rotational rheometer by Lamy Rheology Instruments (Champagne au Mont d’Or, France) for testing the rheological properties of samples. It was equipped with a CP1 Plus thermostat from Lamy Rheology Instruments with Rheomatic Lamy Instruments software. The tests were conducted in a system plate-plate geometry, using the MS CP 2445 Lamy Rheology Instruments measuring system with a diameter of 24 mm (α = 0.45°). Before measurement, the samples were placed in a CLW 53 STD incubator (POL-EKO Aparatura sp.j., Wodzisław Śląski, Poland) at a temperature of 25.0 ± 0.5 °C. After 30 min, the sample was placed on the lower plate of the Peltier effect thermostatic system made of stainless steel, and the upper plate was lowered. The height of the gap between the plates was 50 μm. Excess sample was removed with a spatula. All rheological tests were carried out at 25.0 ± 0.5 °C. For the tested ointments, viscosity was measured at two shear rates, 15 s^−1^and 30 s^−1^, with shear stress measurement in relation to the shear rate (step by step) and a flow curve (flow test and hysteresis loops). Five repetitions were conducted for each experiment, and the mean values of the calculated parameters are presented (n = 5).

#### Flow Curves in Controlled Shear Stress Mode

The analysis was carried out in the tangential stress range 5.0–200.0 s^−1^. The obtained results are presented in the form of shear rate dependence on shear stress as flow curves and also in the form of hysteresis loop for step-by-step test. 

### 3.9. Statistical Analysis

Mean values with standard deviation were calculated and statistically analyzed using the Microsoft Excell package and Statistica (StatSoft, Inc., Kraków, Poland) option: Industrial analysis, experimental design (DOE). The Statistica Pharmaceutical Kit: Statistica “Release Profiles” was used to analyze the results of ACTH release from the ointment bases. The Weibull method. Statistically significant significance at *p* < 0.05 was calculated using Student’s *t*-test.

## 4. Conclusions

Analyzing the results of ACTH release test it can be stated that the degree of ACTH release depends on several factors: the base used, the concentration of ACTH, rheological parameters of ointment. Among the bases used, the most favourable ointment base for preparation of ointment from ACTH was hydrogel ointment (glycerol ointment), whereas the optimal concentration of corticotropin in the ointment was 20 mg/g. The higher the hormone concentration, the greater its amount was released into the acceptor fluid. The process of release at the latest began from ointment at concentration of 15 mg/g and as the concentration increased, more corticotropin was released at specific intervals in relation to ointments with the lowest concentration. The degree and rate of release of ACTH depend on the concentration of the active substance in the ointment and on the rheological properties of semisolid formulations. The addition of hormone causes an increase in viscosity in relation to the unloaded hydrogel itself. At the same time, a further increase in the concentration to 20 mg/g and 25 mg/g results in a increase in viscosity and release but increase in viscosity at 25 mg/g ACTH provide decrease in released amount of ACTH.

Based on a study of corticotropin penetration through the natural membrane that is the skin of the pig’s ear, it was found that ACTH penetrates the skin. The inverse dependence of the amount permeated and the rate of hormone permeation on its concentration was obtained. The most advantageous formulation was the ointment with the lowest concentration (F-7, 15 mg/g), from which the corticotropin penetrated in the highest amounts and the fastest. With increased concentration and increased viscosity the amount of penetrating corticotropin decreased. These results do not confirm the relation obtained from the release test with the use of an artificial cellulose membrane. This fact proves different membrane properties and unpredictable character of processes taking place in the skin. Therefore, it is important to check the behavior of the active substance in simulated in vivo conditions. Based on the obtained results, it was found that there was a possibility of using ACTH in the form of ointments in clinical practice (dermatological diseases, multiple sclerosis or Alzheimer’s disease). Semi-solid dosage forms are a convenient form of skin application for potential patients, and unlike injections, a safe and non-invasive method of therapy.

## Figures and Tables

**Table 1:** The pH values determined for the semi-solid formulations tested (*p* < 0.05).

Formulation	F-Control	F-7 25 mg/g ACTH	F-8 20 mg/g ACTH	F-9 15 mg/g ACTH
**pH Value**	4.96 ± 0.02	6.44 ± 0.02	6.24 ± 0.01	5.99 ± 0.03

**Table 2:** Kinetics release models used to describe the release of ACTH from various formulations.

Formulation	Zero Order	First Order	Higuchi Model	Korsmeyer-Peppas Model
Regression Coefficient R^2^				
F-7 (25 mg/g)	0.969	0.914	0.876	0.996
F-8 (20 mg/g)	0.986	0.695	0.925	0.986
F-9 (15 mg/g)	0.858	0.834	0.728	0.950

**Table 3:** Parameters of ACTH availability from hydrogel ointments.

Formulation	Cumulative Release ACTH Amount [mg/cm^2^]	Availability–AUC (0–6 h) [mg/cm^3^/h^−1^]	Degree of Relative Availability EBA [%]
F-7 (25 mg/g)	4.77 ± 0.47 *	11.393	745.1
F-8 (20 mg/g)	5.23 ± 0.36 *	15.585	1019.3
F-9 (15 mg/g)	0.99 ± 0.27	1.853	100.0
R^2^	0.812	0.683	

* statistically significant difference (*p* < 0.05) with respect to preparation F-9 (15 mg/g ACTH).

**Table 4:** The rate of penetration of ACTH through pig skin from hydrogel ointment taking into account the dependence of the amount permeated to the surface in cm^2^ w on the square root of time (using Higuchi’s model), (n = 5); * *p* < 0.05.

Formulation	Average Release Rate (mg/cm^2^/min^1/2^) ± SD	R^2^
F-7 (25 mg/g)	0.17 ± 0.01 *	0.9958
F-8 (20 mg/g)	0.18 ± 0.04 *	0.9953
F-9 (15 mg/g)	0.22 ± 0.02	0.9966

* Statistically significant difference (*p* < 0.05) with respect to preparation F-9 (15 mg/g ACTH).

**Table 5:** Viscosity and shear stress values of hydrogels F-7–F-9 and F-control, */** statistically significant difference (*p* < 0.05/*p* < 0.01) with respect to formulation F-control).

Formulation	Shear Rate			
	15 s^−1^		30 s^−1^	
	Viscosity [Pa·s]	Shear Stress [Pa]	Viscosity [Pa·s]	Shear Stress [Pa]
Glycerol Ointment (F-control)	6.01 ± 0.26	90.1 ± 11.32	6.08 ± 0.17	182.52 ± 20.91
F-9 (15 mg/g)	7.6 ± 0.185 **	113.89 ± 2.77 **	5.17 ± 0.03 **	158.57± 0.86 **
F-8 (20 mg/g)	9.2 ± 0.17 **	137.71 ± 2.51 **	5.87 ± 0.21	176.17 ± 6.17
F-7 (25 mg/g)	10.0 ± 0.3 **	149.57 ± 4.50 **	6.46 ± 0.07 *	193.95 ± 2.06 *

**Table 6:** Composition of prepared corticotropin ointments (F-1–F-9).

Preparation	Vehicle	ACTH Concentration in 1 M Aqueous Acetic Acid Added to the Vehicle [mg/mL]	Amount of ACTH Added as Lyophilisate in 10.0 Ointment [mg]	Final ACTH Concentration in the Ointment [mg/g]
F-1	Lekobaza Lux	-	250	25
F-2	Lekobaza Lux	-	200	20
F-3	Lekobaza Lux	-	150	15
F-4	Eucerin Ointment I	250	-	25
F-5	Eucerin Ointment I	200	-	20
F-6	Eucerin Ointment I	150	-	15
F-7	Glycerol Ointment ^1^	-	250	25
F-8	Glycerol Ointment ^1^	-	200	20
F-9	Glycerol Ointment ^1^	-	150	15
**Reference Ointment**				
Glycerol Ointment ^1^ (F-control)-wheat Starch (10.0), purified Water (15.0), glycerol 85% (90.0), ethanol 760 g/L (1.0)				
